# *Staphylococcus aureus* in Bovine Mastitis: A Narrative Review of Prevalence, Antimicrobial Resistance, and Advances in Detection Strategies

**DOI:** 10.3390/antibiotics14080810

**Published:** 2025-08-08

**Authors:** Rahima Touaitia, Nasir Adam Ibrahim, Abdelaziz Touati, Takfarinas Idres

**Affiliations:** 1 Department of Natural and Life Sciences, Faculty of Exact Sciences and Natural and Life Sciences, University of Tebessa, Tebessa 12000, Algeria; rahima.touaitia@univ-tebessa.dz; 2 Department of Biology, College of Science, Imam Mohammad Ibn Saud Islamic University (IMSIU), Riyadh 13318, Saudi Arabia; naabdalneim@imamu.edu.sa; 3 Laboratoire d’Ecologie Microbienne, FSNV, Université de Bejaia, Bejaia 06000, Algeria; abdelaziz.touati@univ-bejaia.dz; 4 Laboratory for Livestock Animal Production and Health Research, Rabie Bouchama National Veterinary School of Algiers, Issad ABBAS Street, BP 161 Oued Smar, Algiers 16059, Algeria

**Keywords:** bovine mastitis, *Staphylococcus aureus*, antimicrobial resistance, subclinical mastitis, one Health

## Abstract

Bovine mastitis, particularly that caused by *Staphylococcus aureus*, presents a major challenge to dairy production worldwide due to its economic impact, animal welfare concerns, and zoonotic potential. This narrative review synthesizes current literature on the epidemiology, pathogenesis, resistance patterns, and control strategies related to *S. aureus*-associated mastitis in dairy cattle. It highlights the pathogen’s virulence mechanisms, such as biofilm formation, immune evasion, and toxin production, that facilitate persistent infections. The review compiles global prevalence data, revealing significant geographic variation and disparities between clinical and subclinical cases. Antimicrobial resistance, especially the emergence of methicillin-resistant *S. aureus* (MRSA), is extensively examined alongside resistance gene profiles. Diagnostic approaches, including culture, PCR, MALDI-TOF MS, and AI-based systems, are evaluated for their sensitivity and field applicability. Additionally, the review addresses public health implications, zoonotic risks, and One Health perspectives, culminating in an exploration of prevention strategies, including improved hygiene, vaccination, dry cow therapy, and AI-driven herd management. The findings emphasize the urgent need for integrated surveillance, precision diagnostics, and targeted interventions to mitigate the burden of *S. aureus* mastitis.

## 1. Introduction

Bovine mastitis imposes a substantial economic burden on the global dairy industry, with direct losses attributed to reduced milk yield, treatment costs, and premature culling of infected animals. In southeastern Australia, the annual expenses of mastitis are estimated at AUD 150 million, driven by production losses, veterinary costs, and culling [1,2,3,4]. Globally, annual losses range between USD 19.7 and 32 billion, with subclinical mastitis contributing disproportionately due to undetected infections [5,6]. The disease also compromises animal welfare, causing udder pain, inflammation, and systemic illness, which reduces the quality of life for affected cows [4,6,7].

*S. aureus* is a leading causative agent of bovine mastitis worldwide, implicated in both clinical and subclinical forms. Its prevalence varies geographically: in southeastern Australia, *S. aureus* accounts for 10.6% of clinical and 29.1% of subclinical mastitis isolates [1]. In Ethiopia, a study of 15,000 cows across 46 herds reported a pooled prevalence of 35% for *S. aureus* mastitis, with subclinical cases predominant [8,9]. Similarly, in Central Oromia, Ethiopia, a 73.7% overall mastitis prevalence was observed, with *S. aureus* as the primary pathogen [10]. Regional disparities are evident; in China, *S. aureus* prevalence in clinical mastitis ranges from 10% to 66.6% across studies, influenced by herd management and diagnostic practices [11].

The pathogen’s ability to persist in mammary tissues and evade immune responses exacerbates its global burden. *S. aureus* biofilm formation and antimicrobial resistance (AMR) further complicate efforts to control the infection. In Ethiopia, beta-lactam resistance rates among *S. aureus* isolates reach 68%, linked to widespread antibiotic misuse [8]. Methicillin-resistant *S. aureus* (MRSA) strains, including livestock-associated variants, are increasingly reported, with *mecA*-mediated resistance posing zoonotic risks [12,13]. Despite low overall resistance rates in some regions, such as southeastern Australia, AMR surveillance remains crucial in mitigating therapeutic failures [1].

Diagnostic challenges compound the economic and health impacts of *S. aureus* mastitis. Intermittent bacterial shedding in milk complicates detection, with culture, PCR, and MALDI-TOF mass spectrometry exhibiting variable sensitivity [14,15]. Subclinical infections, often undiagnosed without tools like the California Mastitis Test, contribute to chronic herd infections and sustained transmission [7,10]. These factors underscore the need for enhanced diagnostics and tailored control strategies to address the pathogen’s epidemiological complexity.

This narrative review synthesizes structured data from scientific abstracts to examine the epidemiological aspects of *S. aureus*-associated bovine mastitis. The purpose is to consolidate current knowledge on global prevalence trends, geographic and herd-level distribution patterns, and clinical versus subclinical manifestations. It further explores challenges related to antimicrobial resistance, diagnostic limitations, and control strategies. The review does not interpret experimental or clinical trial data but focuses on synthesizing reported prevalence rates, risk factors, and resistance profiles. By analyzing these compiled findings, the review aims to highlight regional disparities, identify gaps in surveillance, and inform future research and public health interventions targeting *S. aureus* mastitis.

## 2. Literature Search Strategy

This narrative review employed a systematic literature search strategy to synthesize current knowledge on *Staphylococcus aureus* in bovine mastitis, with a focus on prevalence, antimicrobial resistance (AMR), pathogenesis, detection, and control strategies. The search was conducted across three major databases “PubMed, Scopus, and Web of Science Core Collection” spanning the last decade (January 2015 to June 2025).

The search strategy incorporated a comprehensive set of keywords and Boolean operators to capture relevant studies. Core terms included “*Staphylococcus aureus*” OR “*S. aureus*” combined with “bovine mastitis” OR “dairy cow mastitis” OR “udder infection”, and further refined using thematic subqueries: “prevalence” OR “epidemiology”, “antimicrobial resistance” OR “AMR” OR “MRSA”, “biofilm” OR “intracellular invasion” OR “virulence factors”, “detection” OR “diagnosis” OR “MALDI-TOF” OR “PCR” OR “AI”, and “control” OR “prevention” OR “vaccine” OR “One Health”. Example queries aligned with these groupings, such as (“*Staphylococcus aureus*”) AND (“bovine mastitis”) AND (“antimicrobial resistance”) AND (“biofilm formation”).

Studies were included if they met the following criteria: original research, systematic reviews, or meta-analyses reporting data on *S. aureus* prevalence, AMR profiles, virulence mechanisms, diagnostic methods, or control strategies in bovine mastitis; English-language publications or those with English abstracts; and geographic diversity covering global dairy systems. Exclusion criteria removed non-bovine studies (e.g., human or small ruminant focus), articles centered on non-*S. aureus* pathogens, non-peer-reviewed works (e.g., conference abstracts without full data), and publications lacking methodological rigor or statistical validation.

Screening occurred in two stages: an initial assessment of titles and abstracts for topical relevance, followed by a full-text evaluation of retained articles for methodological quality, data granularity, and alignment with the review objectives. To ensure comprehensiveness, reference lists of key publications were hand-searched for additional sources via snowballing. Extracted data encompassed prevalence rates, AMR gene profiles, diagnostic performance metrics, and intervention efficacy, and synthesized thematically to highlight regional disparities, temporal trends, and research gaps.

Study quality was evaluated based on sample representativeness, adherence to standardized diagnostic protocols (e.g., CLSI guidelines for AMR testing), and statistical robustness (e.g., confidence intervals for prevalence estimates). Limitations included potential language bias due to English-only inclusion, uneven geographic representation (e.g., scarce data from Africa and South America), and heterogeneity in diagnostic criteria (e.g., variable somatic cell count thresholds for subclinical mastitis).

## 3. Pathogenesis and Virulence Factors of *S. aureus* in Bovine Mastitis

### 3.1. Overview of Pathogenesis

**Figure 1:**
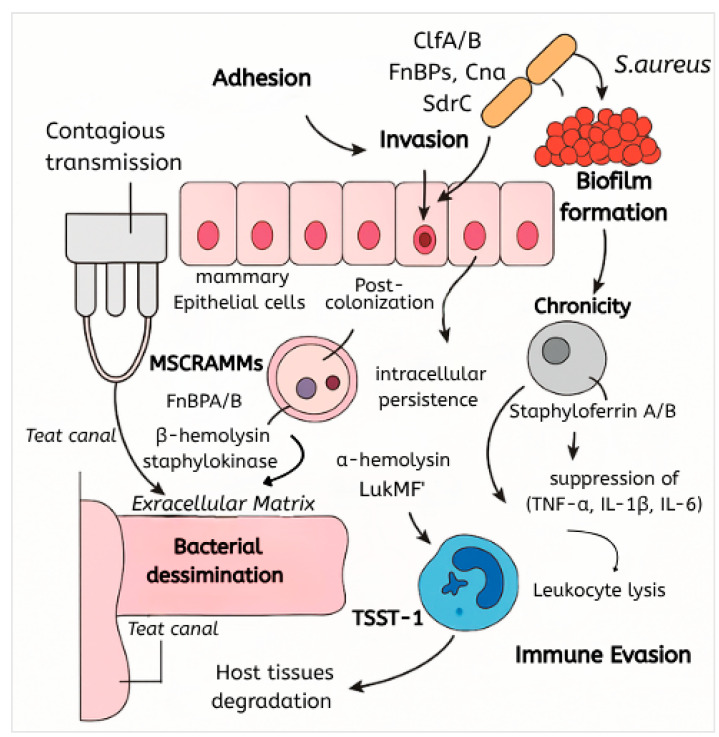
Schematic of *S. aureus* pathogenesis and immune evasion.

*S. aureus* initiates bovine mastitis by invading the mammary gland through the teat canal (Figure 1), often exploiting mechanical damage or compromised teat keratin barriers [7,16]. Contagious transmission during milking facilitates colonization, driven by adhesins, such as fibronectin-binding proteins (FnBPs), clumping factors (ClfA/B), and collagen-binding proteins (Cna) [2,17]. These microbial surface components recognizing adhesive matrix molecules (MSCRAMMs) enable attachment to host extracellular matrix components and mammary epithelial cells [18]. Post-colonization, the pathogen evades immune defenses via intracellular persistence within mammary epithelial cells or phagolysosomal compartments [6,19], alongside suppression of toll-like receptor (TLR)/NF-κB signaling to dampen inflammatory responses [20]. Chronicity is perpetuated by biofilm formation, toxin-mediated tissue damage, and nutrient acquisition systems, such as siderophores (staphyloferrin A/B) and the iron-responsive Isd system, which scavenge essential metals in the nutrient-restricted mammary environment [21,22].

### 3.2. Adhesion and Colonization Mechanisms

Adhesion is mediated by MSCRAMMs, including FnBPA/B, ClfA/B, Cna, and SdrC, which bind host fibronectin, collagen, and other extracellular matrix components [7,22]. Fibronectin-binding proteins (FnBPs) are crucial for initial attachment to the mammary epithelium, while clumping factors facilitate aggregation and biofilm initiation [18]. Collagen adhesin (Cna) enhances tissue specificity, particularly in damaged teat canals [23]. These interactions are foundational for establishing infection and subsequent biofilm development [24].

### 3.3. Toxin Production and Tissue Damage

*S. aureus* secretes cytolytic toxins and superantigens that exacerbate tissue damage and immune dysregulation. Pore-forming alpha-hemolysin disrupts epithelial integrity, while leukocidins, particularly LukMF’ (specific to bovine neutrophils), induce leukocyte lysis, impairing innate immunity [25]. Superantigens, including enterotoxins and toxic shock syndrome toxin-1 (TSST-1), trigger hyperinflammation through excessive cytokine release [20,26]. β-hemolysin and staphylokinase further degrade host tissues, facilitating bacterial dissemination [27,28].

### 3.4. Biofilm Formation and Persistence

Biofilm formation is a central virulence mechanism of *S. aureus*, particularly in chronic bovine mastitis [18,29,30]. These structured microbial communities are embedded in a self-produced extracellular matrix that adheres to host tissues or abiotic surfaces. In the context of the bovine mammary gland, biofilms facilitate bacterial persistence by enhancing resistance to host immune responses and antimicrobial therapies, thereby increasing the risk of chronic and recurrent intramammary infections (IMIs) [31].

The biofilm developmental process in *S. aureus* comprises four sequential stages: initial attachment, accumulation and maturation, maintenance, and dispersal. MSCRAMMs, such as FnBPs, ClfA/B, and Cna, primarily mediate the initial adherence to mammary epithelial surfaces. These proteins facilitate binding to host fibronectin and collagen, enabling bacterial colonization at the infection site [30,31].

Following the attachment, biofilm maturation involves the synthesis of extracellular polymeric substances, especially polysaccharide intercellular adhesin (PIA), which is encoded by the *icaADBC* operon [22]. PIA promotes intercellular aggregation and structural integrity of the biofilm. In some bovine strains, biofilm-associated protein (Bap) further enhances biofilm stability, especially under milk-rich environments. These mechanisms contribute to the robust biofilm phenotype observed in clinical mastitis isolates [24,32].

The accessory gene regulator (*agr*) quorum-sensing system governs biofilm dynamics. During early biofilm development, *agr* is typically repressed, which facilitates matrix accumulation and reduces immune detection [18]. In contrast, the later activation of *agr* promotes the production of dispersal agents, such as phenol-soluble modulins (PSMs) and proteases, allowing bacteria to exit the biofilm and colonize new sites [31]. This regulatory flexibility enables *S. aureus* to persist within the host while maintaining invasive potential. Environmental and host factors play critical roles in modulating biofilm formation. For example, milk components, such as caseins, enhance bacterial adhesion and matrix formation, serving as a nutrient source that supports high biomass production [33]. Notably, sub-inhibitory concentrations of antibiotics can paradoxically induce biofilm formation, complicating treatment strategies [31].

Biofilm formation significantly enhances the virulence of *S. aureus* through several mechanisms. First, the biofilm matrix impedes antibiotic penetration, rendering embedded bacteria up to 1000 times more resistant to treatment compared to planktonic cells [34]. Second, biofilms protect bacteria from phagocytosis and adaptive immune clearance, facilitating persistent colonization. Additionally, biofilms facilitate horizontal gene transfer, enabling the spread of AMR determinants and further complicating herd-level control [35].

Clinically, biofilm-associated mastitis is correlated with elevated somatic cell counts, reduced milk quality, and prolonged infection duration. Recent surveillance studies in dairy herds across Asia and Latin America confirm a high prevalence of strong biofilm-producing *S. aureus* strains, many of which also carry multidrug resistance genes [34,35].

### 3.5. Intracellular Invasion of S. aureus in Bovine Mastitis

*S. aureus* strains that cause bovine mastitis do more than colonize the mammary gland surface; many actively invade and persist inside bovine mammary epithelial cells (BMECs) and innate immune cells, creating protected reservoirs that drive chronic and recurrent infections [18]. Table 1 summarizes the key stages of *S. aureus* intracellular invasion in bovine mammary epithelial cells, highlighting recent mechanistic insights from molecular studies. It outlines bacterial entry mechanisms, subversion of host cellular processes (such as mitophagy), development of antibiotic-tolerant phenotypes, and the consequences for chronic infection and recurrence. These findings identify potential therapeutic targets for controlling intracellular *S. aureus* during mastitis.

### 3.6. Immune Evasion Strategies

Immune evasion involves multiple mechanisms: protein A binds to immunoglobulins, inhibiting opsonization [19], while capsular polysaccharides impede phagocytosis [6]. Intracellular survival within mammary epithelial cells and phagocytes avoids immune clearance [38,39], which is complemented by the suppression of proinflammatory cytokines (TNF-α, IL-1β, IL-6) and the induction of autophagy [20]. Leukocidin LukMF’ directly lyses neutrophils [40], and superantigens disrupt adaptive immunity by overactivating T cells [26]. Additionally, *S. aureus* downregulates host adhesion molecules (ICAM1, VCAM1) and counteracts nutritional immunity by acquiring metals via siderophores and the Isd system, overcoming lactoferrin-mediated iron sequestration [21,28].

## 4. Prevalence of *S. aureus* in Bovine Mastitis

### 4.1. Geographic Distribution

The global prevalence of *S. aureus* in bovine mastitis exhibits substantial regional variation, influenced by management practices and the use of antimicrobials. Table 2 synthesizes data from some studies worldwide on the prevalence of *S. aureus* mastitis in dairy herds, organized by continent and country.

In Africa, Cameroon reported a 67.0% prevalence (201/300 milk samples), while Egypt documented 44.4% (156/352 quarter milk samples), with MRSA detected in 23.0% and 95.0% of isolates, respectively [38,39]. Ethiopia identified *S. aureus* in 19% of 600 quarter milk samples, and Rwanda detected 135 isolates from 1080 quarter milk samples across 80 farms [41,42].

In Asia, Pakistan reported prevalence rates ranging from 30.32% (94/310 subclinical mastitis samples) to 42.5% (85/200 milk samples), with MRSA in 11.7–21.0% of isolates. Bangladesh documented 46.66% *S. aureus* prevalence (56/120 clinical mastitis samples), while Vietnam and Thailand recorded lower rates of 12.0% (48/400) and 4.76% (4/84), respectively [44,49,51]. South Korea detected MRSA in 6.1% (30/488 isolates), with resistance influenced by farm type [61]. Iran reported 24 *S. aureus* isolates from subclinical mastitis, and China identified a 7.0% prevalence (4/57 raw milk samples) in Shanghai [45,46].

European studies revealed a 32.95% herd-level prevalence in northern Italy (based on 88 bulk tank milk samples), 7.79% in Poland (36/462 milk samples), and 46.1% in Romania (150/325 CMT-positive samples), with MRSA detected in 25.0% of Romanian isolates [53,56]. Ireland reported annual prevalence rates of 21.37–25.59% across 7833 milk samples [52].

Mexico documented a 42% prevalence of *S. aureus* in 50 cows with subclinical mastitis in the Americas, while the U.S. reported 3.3% (158/4794 cows) in clinical cases. Canada observed no MRSA in 611 isolates but noted a 46.6% rate of penicillin resistance in clinical mastitis [57,59,60]. Brazil reported *S. aureus* in 66% of staphylococcal isolates from subclinical mastitis (507 quarter milk samples) [62].

### 4.2. Clinical vs. Subclinical Mastitis

*S. aureus* prevalence varies between clinical (CM) and subclinical mastitis (SCM). In Pakistan’s Narowal District, SCM exhibited higher prevalence (45.8%) than CM (37.5%), while Cameroonian MRSA isolates predominantly originated from clinical cases (72.5%) [38,49]. Conversely, a U.S. study linked *S. aureus* to 3.3% of clinical mastitis cases, correlating with elevated somatic cell counts [60]. Southern Italy reported a 59.16% prevalence of *S. aureus* in bulk tank milk from herds with clinical infections, compared to 29.16% for *S. agalactiae* [4].

Subclinical infections predominated in Ethiopia (15% of cases) and Brazil, where *agr*-negative strains with intracellular persistence mechanisms were associated with SCM [41,63]. A meta-analysis of 22 studies found no significant genetic differences in virulence gene prevalence between CM and SCM cases [64].

### 4.3. Herd-Level Risk Factors and Management Practices

Herd management systems have a significant influence on *S. aureus* epidemiology. Semi-intensive systems in Cameroon and conventional farming in Pakistan correlated with higher MRSA prevalence due to poor milking hygiene and antibiotic misuse [38,49]. South Korean organic farms exhibited lower antimicrobial resistance, despite higher isolation rates, which was attributed to the restricted use of antibiotics [61]. Larger Ethiopian farms with poor hygiene had elevated infection rates, while Irish pasture-based systems with seasonal calving showed persistent *S. aureus* infections (61.84% penicillin-resistant strains) [41,52]. Housing systems also played a role: deep litter systems in Italy correlated with higher prevalence than cubicles [4].

Clonal distribution analysis revealed limited diversity in Rwanda, dominated by CC97 (37%) and CC3666 (33%) lineages. In contrast, Brazilian isolates under CC97 displayed conserved spa types (t605, t521) [42,65].

## 5. Resistance Profiles and Commonly Reported Antimicrobials

### 5.1. Resistance Rates

*S. aureus* isolates from bovine mastitis exhibit extensive resistance to β-lactam antibiotics, with penicillin resistance rates ranging from 21.8% in Thailand/Cambodia to 100% in Egypt and Bangladesh [44,46,66,67]. Similarly, ampicillin resistance ranges from 45.74% to 85% in regions, such as Pakistan, Brazil, Iran, and Bangladesh [31,44,46,48]. Methicillin resistance is geographically variable, with MRSA prevalence ranging from 3.09% in China to 100% in Egypt. In contrast, Canadian and Thai/Cambodian studies report no MRSA [44,59,66,67,68].

Tetracycline resistance varies widely, from 7.3% in Thailand/Cambodia to 83.3% in Iran [46,66]. Resistance to erythromycin ranges from 2.6% in Canada to 57% in Bangladesh [44,59]. Multidrug resistance (MDR) is prevalent globally, with 50–83.3% of MRSA isolates resistant to ≥3 antibiotic classes in Pakistan, South Korea, Egypt, and Bangladesh [44,48,67,69]. Resistance to newer cephalosporins (e.g., cefquinome) and vancomycin remains low but is emerging, with vancomycin resistance genes (*vanA*, *vanB*) detected in 60.8–73.9% of Egyptian VRSA isolates [67,70].

### 5.2. Detection of Specific Resistance Genes

The *mecA* gene, which encodes methicillin resistance, is detected in 17.02–100% of *S. aureus* isolates, with a 100% prevalence in Egyptian MRSA and 66.67% prevalence in broader studies [41,48,67,69]. Beta-lactamase gene *blaZ* is prevalent in 23–73% of isolates, with higher frequencies in human-associated strains compared to bovine isolates [48,52,70,71]. Tetracycline resistance genes (*tetK*, *tet*(*38*)) are reported in 46.80% of Pakistani and Irish isolates, respectively (Table 3) [48,52].

Aminoglycoside resistance genes (*ant*(*4′*)*-Ia*, *aac*(*6′*)*-Ie* + *aph*(*2″*)) are prominent in South Korean MRSA (70% and 40%, respectively). In contrast, macrolide resistance genes (*ermC*, *msrA*) and efflux pump genes (*norA*, *lmrS*) are detected at lower frequencies [69,72,73]. Unique findings include the *fosB* fosfomycin resistance gene in U.S. CC133 isolates and phenotypic oxacillin resistance without *mecA* in Brazilian CC97 strains [65,73].

**Table 3:** Regional prevalence of AMR genes in *S. aureus* mastitis isolates (reported as a percentage of isolates positive for the gene).

Continent	Country	*mecA* (*%*)	*blaZ* (*%*)	*tetK* (*%*)
Africa	Egypt	100	NR	NR
	South Africa	NR	NR	NR
Asia	Pakistan	17.02	55.31	46.80
	Pakistan	21	NR	NR
	Pakistan	44.82	NR	NR
	South Korea	6.1	NR	NR
	South Korea	10.7	NR	NR
	Bangladesh	21.42	NR	NR
	Thailand	0	NR	NR
	Thailand	0	NR	NR
	China	3.09	NR	NR
Europe	Germany	0.8	NR	NR
	Ireland	NR	~61.8	NR
North America	USA (Vermont)	0	2.6–4.8	NR
	USA (Maine)	0	NR	NR
	Canada	0	NR	NR
South America	Brazil	0	100	NR
Oceania	New Zealand	NR	23	NR

Key Points

- *mecA* prevalence peaks in Egypt (100%) and Pakistan (17–44.8%), while Thailand, USA, Canada, and Brazil report 0%.
- *blaZ* is highest in Brazil (100%), followed by Pakistan (55.3%) and New Zealand (23%).
- *tetK* is only reported in Pakistan (46.8%); no other regions provide data.
- Critical gaps exist for Africa (outside Egypt) and South America (outside Brazil). Phenotypic resistance often lacks genetic validation.
- NR: not reported

### 5.3. Prevalence and Characteristics of MRSA

**Figure 2:**
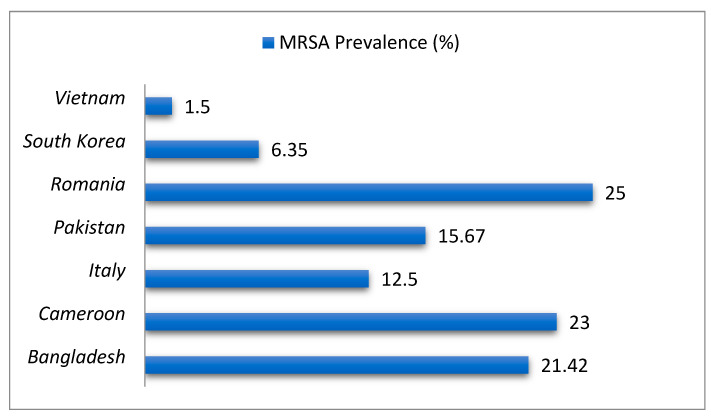
Prevalence of MRSA in milk and *S. aureus* isolates across selected countries.

MRSA strains demonstrate high resistance to β-lactams (90–100%), kanamycin (66.7%), and non-β-lactams, with MDR rates exceeding those of methicillin-susceptible *S. aureus* (MSSA) [48,69]. SCCmec typing reveals geographic dominance: SCC*mec* IV in South Korea (ST72-t324 lineage), SCC*mec* I in Egypt, and SCC*mec* IV/V in Pakistan [47,67,69]. MRSA isolates frequently harbor biofilm-related genes (*clfA*, *icaA/D*) and virulence factors (*pvl*, *tsst-1*, *hlb*, *lukMF*), enhancing persistence and zoonotic potential [44,67,69]. Longitudinal data show rising MRSA prevalence in Germany (2% to 5% over a decade) and phenotypic-genotypic discordance in oxacillin-resistant *mecA*-negative Brazilian isolates [65,70]. Figure 2 shows the prevalence of MRSA in milk and *S. aureus* isolates across selected countries.

### 5.4. Comparative and Cumulative Resistance Patterns

Resistance to β-lactams and tetracyclines is globally widespread, driven by therapeutic overuse, whereas resistance to aminoglycosides and sulfonamides remains comparatively low [46,59]. Regional disparities in resistance gene prevalence (e.g., *blaZ* in 73% of human-associated vs. 2.6% of bovine isolates in Vermont) reflect divergent selection pressures [74]. Declining erythromycin resistance in Germany (53% to 8%) correlates with antimicrobial stewardship, underscoring the impact of targeted interventions [70]. Genotypic–phenotypic discordance, observed in VRSA lacking *vanA*/*vanB* and oxacillin-resistant *mecA*-negative isolates, highlights unresolved resistance mechanisms [65,66].

Cumulatively, recurrent intramammary infections, prophylactic antibiotic use, and horizontal gene transfer contribute to the development of MDR, with urgent needs for alternative therapies and enhanced surveillance to curb the dissemination of resistance [41,75,76].

## 6. Detection and Identification Methods for *S. aureus* in Bovine Mastitis

### 6.1. Conventional Culture and Biochemical Identification

Bacteriological culture remains the cornerstone for detecting *S. aureus* in bovine mastitis. Milk samples are cultured on selective and differential media, including blood agar, mannitol salt agar (MSA), Baird–Parker agar (with egg yolk tellurite), MacConkey agar, Columbia CNA agar, and Chromagar Mastitis, under aerobic incubation at 35–37 °C for 18–48 h [52,57,77,78]. Enrichment steps, such as pre-incubation in tryptic soy broth or nutrient broth with high salt, improve recovery rates [54]. Presumptive identification relies on colony morphology (e.g., golden-yellow pigmentation, hemolysis) and biochemical assays, including Gram staining for Gram-positive cocci, catalase positivity, coagulase activity (via tube or rapid tests), DNase production, and mannitol fermentation [57,61,77]. Chromogenic agars (e.g., Chromagar Mastitis) aid differentiation, while API test systems (e.g., ID 32 STAPH) or automated platforms, like VITEK 2 Compact, provide biochemical profiling for species confirmation [52,61,77]. The California Mastitis Test (CMT) serves as an initial on-farm screening tool for subclinical cases by detecting elevated somatic cell counts [78,79].

### 6.2. Molecular Detection Methods

Molecular techniques enhance specificity and speed. Conventional PCR targeting the *nuc* gene is the gold standard for species confirmation, achieving 100% concordance with phenotypic methods in some studies [80]. Real-time PCR assays, including probe-based systems, detect *nuc* with 100% sensitivity and specificity within 2 h [81]. Multiplex PCR kits (e.g., Mastit 4, bactotype HP3) concurrently identify *S. aureus* and co-pathogens (e.g., streptococci, *Klebsiella pneumoniae*), with sensitivities of 63.3–85.6% and specificities of 93.6–99.2% [45,82]. Resistance genes (*mecA* for methicillin resistance, *vanA*, *blaZ*) and virulence factors (*clfA*, *fnbA*, *pvl*, *icaD*, *Hla*) are detected via PCR or SCC*mec* typing [61,74,83]. Recombinase-aided amplification (RAA) coupled with lateral flow dipsticks (LFD) enables rapid (<45–85 min) field-deployable detection of *nuc* at sensitivities as low as 60 fg DNA [45]. Whole-genome sequencing (WGS) and *spa* gene typing provide strain-level resolution (e.g., ST1, ST97) and comprehensive virulence/resistance profiling [73,84]. Fluorescence in situ hybridization (FISH) with the *Sau* 16S69 probe visualizes *S. aureus* aggregates in tissues [85].

### 6.3. Automated and Commercial Diagnostic Systems

Automated systems streamline workflows. Matrix-assisted laser desorption/ionization time-of-flight mass spectrometry (MALDI-TOF MS) achieves rapid (<24 h), accurate species identification (confidence scores > 2.0). However, differentiation from closely related species (e.g., *S. rostri*) requires updated databases [2,86]. The Bacticam AI-based image classifier automates the analysis of bacterial growth on SELMA + multi-agar plates, achieving a sensitivity of over 85% and a specificity of over 91.9% compared to MALDI-TOF [87]. Commercial qPCR kits (e.g., Mastitis 4E) demonstrate high sensitivity (≥0.95) and specificity (≥0.99) for bulk milk testing [4]. Lateral flow immunoassays (LFIA) targeting EF-Tu protein offer rapid results (15 min) but require high bacterial loads (≥10^6^ CFU/mL) [88]. Gold nanoparticle-enhanced ELISA improves sensitivity (97%) for antigen detection in milk, outperforming conventional ELISA [40]. The CombiFoss 7 DC integrates somatic cell analysis but relies on complementary culture for pathogen-specific identification [89].

### 6.4. Comparative Performance and Validation

As described in Table 4, culture methods exhibit variable sensitivity (50–95.5%) and specificity (79.0–91.4%), with improvements after pre-incubation [54,82]. PCR enhances detection rates by 9–18% over culture alone but faces cost and infrastructure barriers in resource-limited settings [41,90]. Real-time PCR demonstrates superior specificity (97.0–97.6%) compared to culture (89.1%) but lower sensitivity (63.3–78.1%) in pooled samples [82]. MALDI-TOF MS reduces turnaround time but cannot detect resistance genes [86]. The duplex RAA-LFD assay exhibits high concordance (κ > 0.92) with reference methods and detects *S. aureus* within 6 h post-enrichment [45]. Commercial qPCR kits surpass culture in specificity for contagious pathogens, while nanoparticle-enhanced ELISA reduces reliance on culture [4,40].

## 7. Application of Artificial Intelligence in the Diagnosis and Management of *S. aureus*-Associated Bovine Mastitis

### 7.1. AI-Assisted Detection of S. aureus

**Figure 3:**
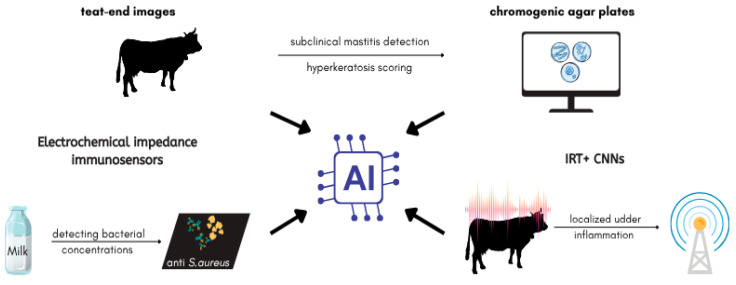
AI-assisted mastitis monitoring system overview.

Artificial intelligence enhances diagnostic precision for *S. aureus*-associated mastitis through multimodal approaches (Figure 3). Electrochemical impedance immunosensors functionalized with anti-*S. aureus* antibodies, combined with decision tree classifiers, achieved 95% accuracy in detecting bacterial concentrations in milk samples, enabling on-farm identification of subclinical infections [91]. Infrared thermography (IRT) paired with convolutional neural networks (CNNs) localized udder inflammation via temperature differentials, though environmental variability reduced sensitivity to 67% in field conditions [92,93]. Deep learning models trained on 16,000 teat-end images automated hyperkeratosis scoring, aiding in the detection of subclinical mastitis [94]. Automated image analysis of chromogenic agar plates improved *S. aureus* identification in milk samples after 24-h incubation, though sensitivity gaps persisted for low-prevalence pathogens [95].

### 7.2. Predictive Modeling for Mastitis Risk and Herd Monitoring

Machine learning (ML) models integrate genomic, sensor, and management data to forecast *S. aureus* mastitis risks. Explainable AI (XAI) methods, including SHapley Additive exPlanations (SHAP), identified immune-related genomic markers (e.g., 204,642 SNPs) linked to mastitis susceptibility in Holstein cows, achieving 65% accuracy in classifying genetic risk [96]. Random Forest (RF) and Extreme Gradient Boosting (XGBoost) algorithms analyzed time-series sensor data (e.g., milk conductivity, yield) to predict the onset of clinical mastitis with 85% recall, although specificity varied (31–62%) [97]. Herd-level models incorporating somatic cell counts (SCC), milk yield, and climatic variables predicted mastitis outbreaks in buffalo herds with 76% accuracy, albeit with suboptimal sensitivity (67%) [98]. Network models simulating cattle movement and farm contact data further supported herd-level assessments of *S. aureus* transmission risk [99].

### 7.3. AI-Enhanced Treatment and AMR Management

AI-driven platforms address *S. aureus* AMR and optimize therapy. Machine learning classifiers applied to MALDI-TOF mass spectrometry data achieved 96.8% accuracy in predicting MDR *S. aureus* strains and 97.5% accuracy for benzylpenicillin resistance, using ribosomal and DNA-binding protein biomarkers identified in 82 isolates [100]. AI platforms that correlate protein–protein interaction networks with resistance mechanisms enable phenotype-guided antimicrobial selection, reducing the empirical use of broad-spectrum antibiotics [101]. Digital twins that integrate real-time sensor data (e.g., udder temperature, feeding behavior) dynamically update treatment recommendations, although pathogen-specific workflows require further development [102]. ML models also guided selective dry cow therapy (SDCT) by identifying cows that required antimicrobial intervention, although “black-box” limitations hindered farmer adoption [103].

### 7.4. Challenges and Future Directions

The integration of AI in managing *S. aureus*-associated bovine mastitis faces significant hurdles, primarily stemming from dataset limitations. Current models often rely on small, regionally constrained training datasets, which undermines their robustness and generalizability. For instance, Beck et al. (2024) trained anomaly-detection algorithms on only (n = 58) metagenomic milk samples, which risks overfitting and fails to capture global pathogen diversity [101]. Similarly, genomic susceptibility studies using whole-genome sequences from 52 cows struggled with biological variability, highlighting the “*p* >> n” problem (predictors far exceeding the number of samples) in complex trait prediction [96].

Adoption barriers further obstruct practical implementation. High costs for sensors, sequencing, and computational infrastructure limit accessibility, particularly for small-scale farms [104]. Compounding this, low farmer tech-literacy complicates interaction with AI interfaces, as seen in studies where complex data management requirements reduced user engagement [105]. Crucially, the “black-box” nature of AI models erodes stakeholder trust; Rowe et al. (2025) documented veterinarian skepticism toward ML-driven treatment recommendations due to the opacity of decision pathways [103].

Addressing these challenges necessitates rigorous validation frameworks. Multi-herd trials are crucial for evaluating generalizability across different breeds, climates, and management systems. For example, teat-end hyperkeratosis classifiers trained on images from two farms require validation in diverse geographical settings to confirm utility [94]. Similarly, MALDI-TOF resistance predictors (with 96.8% accuracy) were used on isolates from single countries, necessitating cross-border studies to verify their global applicability [100]. Longitudinal real-world testing is equally critical; early-detection algorithms (e.g., Random Forest models with 85% recall) must be assessed over extended periods to ensure durability against pathogen evolution [97].

Future advancements should prioritize federated learning to pool data across institutions without compromising privacy, enhancing dataset representativeness [106]. XAI techniques, such as SHAP value visualizations, can demystify model logic and rebuild stakeholder trust [96]. Finally, establishing open-access, multi-herd consortia will standardize validation protocols and accelerate the translation of AI innovations into herd-specific solutions [107].

## 8. Public Health Implications of *S. aureus* from Bovine Mastitis

### 8.1. Zoonotic Transmission and Occupational Exposure

*S. aureus* from bovine mastitis poses significant zoonotic risks through direct occupational exposure to infected cattle, contaminated milk, or dairy environments. Canadian studies demonstrated that bovine-derived strains invade and kill human intestinal cells (Caco-2) and cause lethal infections in *Caenorhabditis elegans*, highlighting cross-species pathogenicity despite lacking human adaptation genes [108]. Molecular epidemiological evidence from Tanzania, Colombia, and Pakistan confirmed bidirectional transmission, with genotypic similarities in erythromycin resistance genes (*ermA*, *ermB*, *ermC*) between bovine mastitis isolates and farm workers’ nasal swabs [109,110,111]. Environmental reservoirs, such as hock skin in Dutch herds and milking equipment in Brazilian farms, further amplify occupational risks, particularly with multidrug-resistant strains [112,113]. Poor hygiene practices, inadequate protective measures, and prolonged bacterial shedding in subclinical infections exacerbate colonization risks for dairy workers [111,114].

### 8.2. Foodborne Transmission and Enterotoxin-Related Risks

Raw milk contaminated with enterotoxigenic *S. aureus* strains, including MRSA variants, represents a critical food safety concern. Enterotoxin genes (*sea*, *seb*, *sed*, *seg*, *tst*) were identified in isolates from India, Tanzania, and Kazakhstan, with biofilm-forming strains enhancing toxin persistence in dairy products [109,115,116]. Subclinical mastitis in cows contributes to undetected pathogen shedding, particularly in regions with high consumption of raw milk. MRSA genotype B (GTB) in raw milk cheese highlights the risks associated with unprocessed dairy products [45]. While pasteurization mitigates risks, interventions, like geraniol (a natural antimicrobial) and bacteriophage therapy, show promise in reducing contamination and antibiotic residues [84,117].

### 8.3. MRSA Strains and Human Health Threats

MRSA from bovine mastitis carries *mecA* and exhibits resistance to β-lactams, tetracyclines, and lincomycin. Canadian MRSA isolates invaded human cells and resisted intracellular antibiotic clearance, raising concerns about treatment failures [108]. Strains from Argentina (ST83, *spa* t002) and China (*S. equorum*, *S. saprophyticus*) highlight zoonotic risks, with virulence genes (*hlg*, *pvl*, *tsst-1*) exacerbating pathogenicity [118,119]. Indian MRSA isolates demonstrated 100% resistance to cefoxitin and 91.3% to oxacillin, alongside biofilm-forming capabilities [115]. Synergistic therapies, such as red ginseng extract, and eradication programs in Switzerland emphasize the need for biosecurity and antibiotic stewardship to curb AMR dissemination [84,120].

### 8.4. One Health Interventions and Surveillance

**Figure 4:**
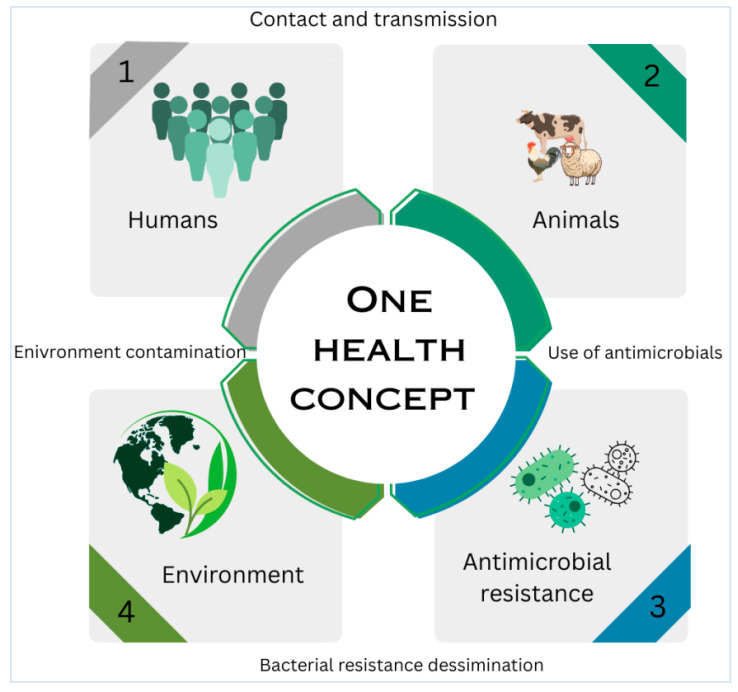
One Health conceptual framework showing zoonotic transmission routes.

Integrated One Health strategies are crucial for addressing zoonotic transmission (Figure 4), AMR spread, and food safety risks. Recommendations include enhanced antimicrobial stewardship, rapid diagnostics (e.g., nanopore metagenomics), and alternatives, like phage endolysins or graphene oxide, to reduce antibiotic reliance [121,122]. Surveillance of extramammary reservoirs (e.g., hock skin, milking equipment) and improved milking hygiene are essential to interrupt transmission cycles [112,123]. Successful eradication programs in Switzerland and Colombia emphasize biosecurity, segregated milking, and farmer education [111,120].

## 9. Prevention and Prophylaxis of *S. aureus* in Bovine Mastitis

*Staphylococcus aureus* mastitis remains a persistent global challenge in dairy production, characterized by significant economic losses, chronic infections, and the evolution of antimicrobial resistance. Practical control demands integrated, multi-faceted strategies due to the pathogen’s capacity for immune evasion, biofilm formation, intracellular persistence, and antigenic diversity. Table 5 synthesizes evidence-based global control approaches.

### 9.1. Hygienic and Management Practices

Pre- and post-milking teat disinfection is a cornerstone of *S. aureus* control. Polyhexamethylene biguanide (PHMB)-based teat disinfectants form a protective antimicrobial barrier, achieving >4-log reduction of *S. aureus* and residual efficacy for 12 h, outperforming traditional iodine-based products [124]. Silver (AgNPs) and copper nanoparticles (CuNPs) at 0.78 mg/L reduce *S. aureus* by 95–97%, disrupting biofilms and inducing bacterial oxidative stress [125]. Bacteriocin-producing lactic acid bacteria (LAB), such as *Lactococcus cremoris* FT27, demonstrate efficacy comparable to that of chemical disinfectants through nisin A production [126]. Post-milking teat dipping reduces new intramammary infections (IMIs) by 62% in field trials [127]. Environmental hygiene measures, including daily lime application, clean straw bedding, and cubicle maintenance, lower bacterial exposure. Automated milking systems (AMS) require tailored protocols, supported by farmer education programs [128]. Improved teat and udder conformation in indigenous breeds correlates with a reduced prevalence of subclinical mastitis (SCM), highlighting the genetic selection benefits. Strict aseptic techniques during dry-off, combined with PCR-based milk monitoring, enable timely interventions [4].

### 9.2. Vaccination and Immunoprophylaxis

Vaccination against *S. aureus* mastitis remains challenging due to antigenic variability, biofilm formation, and transient immune responses. Experimental vaccines targeting *Staphylococcus chromogenes* surface proteins (SCSP) reduced subclinical mastitis incidence by 90% at cow and quarter levels, whereas *S. aureus* surface protein (SASP) vaccines showed non-significant protection (48%) [129]. A trivalent inactivated vaccine (*S. aureus*, *E. coli*, *M. bovis*) reduced clinical mastitis signs and milk bacterial counts to undetectable levels within 96 h post-challenge in heifers [130]. Commercial bacterins and autogenous vaccines yield modest reductions in clinical severity but fail to alter the incidence of infection or shedding [131]. Novel approaches, including live-attenuated mutants (e.g., *vraG*-deleted strains) and nanogel-based formulations, aim to enhance Th1/Th17 immunity. Despite progress, no universal vaccine matches the efficacy of hygiene practices [132].

### 9.3. Antimicrobial Prophylaxis and Dry Cow Therapy

Dry cow therapy (DCT) with antibiotics remains critical for controlling subclinical *S. aureus* infections, although cure rates are modest (30–50%) due to intracellular persistence [128]. Selective DCT, guided by somatic cell count (SCC) thresholds (<200,000 cells/mL), reduces antibiotic use by 20–60% without compromising udder health. However, 11.8% of cows receiving internal teat sealants (ITS) alone harbored persistent *S. aureus* infections, particularly high-yielding cows (>15 kg/day), which exhibit elevated somatic cell counts (SCC) in early lactation. Combining ITS with antibiotics during the dry period reduces early lactation SCC and IMIs compared to ITS alone, which increases IMI odds by 6- to 7-fold [133,134]. *Lactobacillus plantarum* CM49 also shows broad antagonistic activity against *S. aureus* [135].

### 9.4. Selective Treatment Protocols

Tailored protocols integrating SCC trends, bacterial cultures, and algorithm-based decision support improve diagnostic accuracy for selective DCT. Segregation of infected cows delays *S. aureus* transmission and lowers treatment costs. Herds employing PCR pooling for routine screening report reduced antimicrobial use and improved udder health. Culling chronically infected animals and optimizing dry period management further reduce early lactation SCC. In Irish herds with high *S. aureus* prevalence (19.7% herd-level IMI), selective DCT efficacy depended on herd-specific management factors [4,133,136].

### 9.5. Resistance Concerns Linked to Preventive Antimicrobial Use

High beta-lactam resistance rates in *S. aureus* isolates, particularly to penicillin (75%), amoxicillin (67%), and cephalosporins (57%), are driven by beta-lactamase production (via the *blaZ* gene) and biofilm-mediated tolerance [8]. In Ethiopia, *S. aureus* isolates showed a pooled beta-lactam resistance prevalence of 35% (95% CI: 31–41%), whereas non-aureus staphylococci (NAS), such as *S. chromogenes*, harbored *blaZ* at a 22% prevalence. Molecular typing and genomic surveillance are crucial for identifying resistant genotypes and informing stewardship practices [137]. Overuse of antibiotics in non-targeted DCTs exacerbates resistance, emphasizing the need for farmer education and the development of selective protocols [11].

### 9.6. Herd-Level Outcomes and Program Efficacy

Integrated programs that combine hygiene, early diagnosis, and culling reduce the prevalence of *S. aureus* and associated economic losses. In Bavaria, a 10-year study documented a decline from 26% (in 2014) to 15% (in 2023) in culture-positive samples [127]. Herds implementing post-milking disinfection and cubicle cleaning twice daily achieved lower SCC. Modeling supports a “bang-bang” strategy, where initial low cow mixing is followed by phased productivity increases to balance biosecurity and output [136]. Welfare scoring systems correlate lower *S. aureus* prevalence with higher welfare outcomes. Intensive systems reported a higher prevalence of mastitis (39.5%), underscoring the need for robust hygiene [9,133].

## 10. Future Directions

Geographic and molecular surveillance gaps hinder a comprehensive understanding of *S. aureus* mastitis dynamics. Regional disparities in capsule genotype distribution (e.g., CP8) and AMR patterns, such as elevated tetracycline resistance in Jordan and ciprofloxacin resistance in Bangladesh, necessitate expanded global surveillance to address zoonotic risks and region-specific AMR trends [138,139]. Concurrently, molecular characterization of virulence factors, including ferroptosis-related genes (HMOX1, SAT1), immune pathways (TLR2, NF-kappa B), and strain-specific determinants (*adlb*, *agr* systems, MSCRAMMs), remains incomplete. Advanced genotyping (e.g., spa t2873, CC8/CC97 strains) and WGS are critical to elucidate transmission dynamics, biofilm-associated genes, and conserved antigen targets for vaccine development [63,84,140,141,142].

Subclinical mastitis caused by multidrug-resistant *S. aureus* in riverine buffaloes is understudied despite the high prevalence of virulence genes. Prioritizing this form through improved diagnostic accuracy and tailored interventions is essential to mitigate undetected transmission and economic losses [139].

Standardization of methodologies and diagnostic optimization is imperative for field applicability. Refining duplex RAA-LFD and AuNPs-based ELISA for sensitivity, alongside standardizing phage therapy production protocols, will enhance reproducibility [40,45,143]. Parallel advancements in AI-driven classifiers (e.g., Bacticam), lateral flow immunoassays (LFIA) with enhanced antibody specificity, and portable biosensors for real-time detection necessitate the expansion of training datasets and harmonized sample handling protocols [54,88].

Therapeutic innovation must address the resilience of biofilms and intracellular persistence. Promising strategies include phage therapy formulations, anti-biofilm agents (e.g., gemini quaternary ammonium salts), NLRP3 inflammasome inhibitors, and probiotics derived from non-aureus staphylococci (NAS). Vaccine development should prioritize adhesins, hemolysins, and biofilm components to disrupt host-pathogen interactions. [51,85,144,145].

AMR management requires region-specific AMR profiling and stewardship programs guided by molecular resistance gene tracking. Policies that enforce prudent antibiotic use, farmer education on biosecurity, and alternatives, such as phage biocontrol, are vital to curb the escalation of resistance [47,138,139,146,147].

Longitudinal and herd-level studies are crucial for validating the efficacy of mastitis control programs, particularly their environmental impacts (e.g., greenhouse gas emissions) and long-term effects on milk yield. Integrating genetic markers into breeding programs may enhance herd resilience against pathogen-specific mastitis [4,148,149].

## 11. Conclusions

*S. aureus* remains a pervasive and economically burdensome pathogen in bovine mastitis worldwide, characterized by its virulence arsenal, diagnostic challenges, and AMR. This narrative synthesis underscores substantial geographic variability in prevalence, resistance profiles, and diagnostic efficacy, reflecting disparities in herd management practices, biosecurity measures, and surveillance infrastructure. The pathogen’s ability to persist intracellularly, form biofilms, and evade immune responses contributes to chronic and subclinical infections that complicate early detection and control.

The widespread detection of MDR and MRSA strains in dairy herds across continents underscores the urgent need for targeted AMR stewardship and genomic surveillance. Diagnostic advances, including molecular assays, AI-enhanced platforms, and rapid biosensors, offer promising avenues for early and precise detection but require standardization and resource adaptation for widespread adoption.

From a One Health perspective, the zoonotic potential of *S. aureus*, particularly MRSA, through occupational exposure and foodborne transmission, represents a significant public health concern. Integrating antimicrobial stewardship, farmer education, hygienic milking practices, and selective dry cow therapy remains critical to mitigate infection risks and curb the dissemination of resistance.

Future interventions must prioritize vaccine development targeting conserved virulence determinants, the deployment of AI-driven predictive tools for mastitis management, and longitudinal studies assessing environmental, genetic, and herd-level dynamics. A coordinated global response, anchored in robust surveillance, translational research, and policy harmonization, is essential to contain the spread of *S. aureus*-associated bovine mastitis and safeguard both animal and public health.

## Figures and Tables

**Table 1:** Mechanisms of intracellular invasion and persistence of *S. aureus* in bovine mastitis.

Stage of Intracellular Pathogenesis	Recent Mechanistic Findings
Adhesion and entry	Invasion is triggered when fibronectin-binding proteins A/B (FnBPs) on the bacterial surface bridge soluble fibronectin to host α5β1-integrins, activating focal-adhesion kinase and actin remodeling that “zipper” *S. aureus* into BMECs. High-prevalence mastitis lineages (e.g., CC97, CC151) show enriched *fnbA/B* expression and an enhanced internalization capacity [18].
Early intracellular phase	Once inside endocytic vacuoles, *S. aureus* inhibits lysosomal acidification and downregulates mTORC1 nutrient signaling, reducing amino acid import and milk protein synthesis, thereby weakening host tissue functions [36].
Subversion of host quality-control pathways	Two complementary studies have shown that bovine mastitis isolates induce mitophagy to eliminate mitochondria-derived reactive oxygen species (ROS) that would otherwise kill the bacteria. One group linked this effect to the upregulation of the PINK1/Parkin mitophagy pathway in bovine mammary epithelial cells (BMECs) [36]. Another independent study demonstrated a similar mechanism in macrophages, involving the HDAC11/IL-10 signaling axis, which promoted the intracellular survival of *S. aureus* [37].
Persistence & antibiotic tolerance	Nutrient limitation and oxidative stress inside cells select for small-colony variants (SCVs) with slowed metabolism, low membrane potential, and high tolerance to β-lactams and aminoglycosides, explaining frequent treatment failures [18]
Escape or reseeding	Periodic host-cell lysis or exocytosis releases bacteria back into milk ducts, reseeding the udder and neighboring quarters, which clarifies why culling or dry-cow therapy is often required for eradication [36]

**Table 2:** Some reported prevalence of *S. aureus* mastitis in dairy herds.

Country	Total Samples Analyzed	Sample Type	Prevalence of *S. aureus* (%)	Type of Mastitis	Reference
Africa					
Cameroon	300	Individual cow milk	67.0	Clinical and Subclinical	[38]
Egypt	352	Individual cow milk	44.4	Clinical and Subclinical	[39]
Egypt	200	Pooled milk	0.39	Subclinical	[40]
Ethiopia	600	Quarter milk	0.19	Clinical and Subclinical	[41]
Rwanda	1080	Quarter milk	12.5	Not specified	[42]
Asia					
Iraq	50	Individual cow milk	66.0	Clinical and Subclinical	[43]
Bangladesh	120	Individual cow milk	46.66	Clinical	[44]
China	57	Raw milk	7.0	Not Specified	[45]
Iran	200	Individual cow milk	12	Not specified	[46]
Pakistan	173	CMT-positive milk	40.0	Subclinical	[47]
Pakistan	310	Raw milk	30.32	Subclinical	[48]
Pakistan	200	Individual cow milk	42.5	Clinical and Subclinical	[49]
Thailand	84	Lactating cow milk	4.76	Clinical and Subclinical	[50]
Vietnam	400	Raw milk	12.0	Clinical and Subclinical	[51]
Europe					
Ireland	7.833	Milk samples from herds	21.37–25.59 (annual)	Clinical	[52]
Italy	88	Bulk tank milk	32.95% (herd-level)	Not Specified	[53]
Italy	120 herds (BTM samples)	Bulk tank milk	59.16 (herd-level)	Clinical and Subclinical	[4]
Poland	100	Milk	22.0	Clinical and Subclinical	[54]
Poland	462	Composite udder milk	7.79	Clinical and Subclinical	[55]
Romania	325	CMT-positive milk	46.1	Clinical and Subclinical	[56]
Americas					
Mexico	50	Individual cow (CMT)	42 (cow-level)	Subclinical	[57]
Uruguay	191	Herd isolates	81.1	Clinical and Subclinical	[58]
Canada	8.957	Clinical milk samples	15.0	Clinical	[59]
USA	5.703	Individual cow milk	3.3	Clinical and Subclinical	[60]

Quarter milk: Milk collected from individual quarters of a cow’s udder. Raw milk: Unprocessed milk obtained directly from lactating cows. Bulk tank milk (BTM): Milk pooled from multiple cows in a storage tank. CMT-positive milk: Samples identified as mastitis-positive via the California Mastitis Test. Composite udder milk: Combined milk from all quarters of a cow’s udder. Herd isolates: Samples derived from entire herds rather than individual cows.

**Table 4:** Comparative Analysis of Diagnostic Methods for *S. aureus* Mastitis.

Technique	Sensitivity	Specificity	Cost	Turnaround Time	Field Applicability	Key Insights
Culture	Moderate (50–89.7%; ↓ in mixed infections)	Moderate–High (challenges in CNS differentiation)	$ Low	Days (24–72 h)	★★☆☆☆ (Lab-dependent)	Standard method; sensitivity improves after competitive pathogen elimination. Limitations for non-culturable strains
PCR	High (near 100%; ↑ with pre-incubation)	High (species-specific)	$$$ High	Hours (4–24 h)	★★☆☆☆ (Lab equipment)	Detects virulence/resistance genes (*nuc*, *mecA*); requires enrichment for low loads. Higher cost limits field use.
qPCR	Very High (≥95%)	Very High (≥99%)	$$$ High	Hours (2–6 h)	★★☆☆☆ (Equipment needed)	Probe-based assays achieve 100% specificity; ideal for bulk milk screening. Cost-prohibitive in resource-limited settings.
RAA-LFD	Very High (60 fg DNA; 1.78 × 10^3^ CFU/mL)	Very High (no cross-reactivity)	$ Low	Minutes (45–85 min)	★★★★★ (Portable, dipstick)	Rapid, equipment-free detection; ideal for field use. Detects *S. aureus* in spiked milk after 6-hr enrichment.
MALDI-TOF	High (score > 2.0)	High (species ID)	$$$ High (instrument)	Hours (post-culture)	★★☆☆☆ (Lab-based)	Rapid ID post-culture is ineffective for detecting AMR. Limited by database accuracy.
AI-Based Systems	Variable (>85% for *S. aureus*; ↓ for rare pathogens)	Very High (91.9–99.1%)	$$ Moderate	Days (24–48 h)	★★★☆☆ (On-farm/Lab)	Automated colony ID (e.g., Bacticam); struggles with mixed infections and transport delays. WGS offers high accuracy but costly/non-portable.

Legend: ↑: increased or enhanced performance. ↓: decreased or limited performance. $: Low (accessible in resource-limited settings). $$: moderate cost. $$$:High (prohibitive for routine use in low-resource areas). ★★☆☆☆: Low to moderate field applicability (lab equipment or skilled handling required), ★★★☆☆: Moderate field applicability (limited portability or requires minimal equipment), ★★★★★: No lab needed (e.g., dipstick tests).

**Table 5:** Global control strategies for *S. aureus* mastitis.

Strategy Category	Specific Approach	Key Findings/Efficacy
Hygiene Practices	Pre-/post-milking teat disinfection (e.g., PHMB, iodine, LAB-based dips)	- PHMB disinfectants achieve >4-log reduction of *S. aureus* with 12-h residual efficacy. - Post-milking dipping reduces new infections by 62%. - LAB-based dips (e.g., *L. cremoris*) match iodine efficacy.
	Environmental management (bedding, housing, cubicle hygiene)	- Clean straw bedding + daily lime application reduces bacterial exposure. - Twice-daily cubicle cleaning lowers early lactation SCC. - Deep litter housing increases risk vs. cubicles.
	Milking protocols (gloves, aseptic techniques, machine sanitation)	- Improper teat disinfection increases risk (OR = 54.83). - Bucket milking (OR = 9.16) and lack of individual towels elevate transmission risk.
Vaccination	Whole-cell/bacterin vaccines (e.g., *S. aureus* surface proteins, toxoids)	- Modest reduction in clinical severity but limited impact on infection incidence. - Antibody responses (IgG2) wane after 4 months; poor correlation with protection.
	Multivalent vaccines (e.g., *S. aureus* + *E. coli* + *M. bovis*)	- Trivalent vaccine reduces bacterial counts to undetectable levels within 96 h post-challenge. - Accelerates SCC normalization in heifers.
	Novel approaches (live-attenuated mutants, DNA/nanogel vaccines)	- Target biofilm/QS systems (e.g., *vraG*-deletion mutants). - Aim to induce Th1/Th17 cellular immunity for intracellular clearance.
Dry Cow Therapy (DCT)	Selective DCT (antibiotics + ITS for infected cows; ITS alone for low-SCC cows)	- Reduces antibiotic use by 20–60% without udder health compromise. - ITS alone increases IMI odds 6–7× vs. antibiotic + ITS (due to *S. aureus* persistence).
	Adjuncts: Internal teat sealants (ITS)	- Critical for preventing new infections during dry period. - Efficacy varies by herd infection pressure; high-risk herds require antibiotics + ITS.
	Non-antibiotic alternatives (probiotics, nanoparticles)	- *B. subtilis* inhibits biofilm via QS interference (*agrA*, *ica* downregulation). - Ag/Cu nanoparticles disrupt biofilms (95–97% pathogen reduction at 0.78 mg/L).
AI-Driven Interventions	Algorithm-based DCT decision support	- Combines SCC with bacterial cultures to improve accuracy. - Reduces under-treatment of low-SCC *S. aureus* infections.
	PCR pooling for herd surveillance (e.g., 10-cow pools)	- Cost-effective screening in automated milking systems (AMS). - Sensitivity: 63–78% in 10-cow PMQS pools (specificity > 97%).
	Optimal mixing/segregation modeling (e.g., “bang-bang” strategy)	- Early reduced mixing delays transmission and lowers treatment costs. - Balances productivity with infection control via quarantine/segregation.
	Bayesian latent class models for diagnostics	- Accounts for imperfect gold standards (e.g., bacteriological culture). - Enhances reliability of pooled-sample PCR testing.

Key Abbreviations: PHMB: Polyhexamethylene biguanide. LAB: Lactic acid bacteria. ITS: Internal teat sealant. SCC: Somatic cell count. IMI: Intramammary infection. QS: Quorum sensing. OR: Odds ratio. PMQS: Pooled post-milking quarter samples. AMS: Automated milking systems.

## Data Availability

The data presented in this study are available within the article. Raw data supporting this study are available from the corresponding author upon reasonable request.

## Abbreviations

The following abbreviations are used in this manuscript:

AMR	Antimicrobial Resistance
AI	Artificial Intelligence
BTM	Bulk Tank Milk
CFU/mL	Colony Forming Units per Milliliter
CMT	California Mastitis Test
CM	Clinical Mastitis
CNN	Convolutional Neural Network
DCT	Dry Cow Therapy
EF-Tu	Elongation Factor Thermo-unstable
ELISA	Enzyme-Linked Immunosorbent Assay
IRT	Infrared Thermography
ITS	Internal Teat Sealant
LFD	Lateral Flow Dipstick
LFIA	Lateral Flow Immunoassay
MALDI-TOF MS	Matrix-Assisted Laser Desorption/Ionization Time-of-Flight Mass Spectrometry
MDR	Multidrug Resistance
MRSA	Methicillin-Resistant *Staphylococcus aureus*
MSCRAMMs	Microbial Surface Components Recognizing Adhesive Matrix Molecules
MSSA	Methicillin-Susceptible *Staphylococcus aureus*
NAS	Non-aureus Staphylococci
PCR	Polymerase Chain Reaction
PIA	Polysaccharide Intercellular Adhesin
qPCR	Quantitative Polymerase Chain Reaction
RAA	Recombinase-Aided Amplification
RF	Random Forest (Machine Learning Algorithm)
SCC	Somatic Cell Count
SCCmec	Staphylococcal Cassette Chromosome mec
SDCT	Selective Dry Cow Therapy
SHAP	SHapley Additive exPlanations (Explainable AI Technique)
SNP	Single Nucleotide Polymorphism
ST	Sequence Type (used in molecular typing)
TSST-1	Toxic Shock Syndrome Toxin-1
WGS	Whole-Genome Sequencing
XAI	Explainable Artificial Intelligence
XGBoost	Extreme Gradient Boosting (Machine Learning Algorithm)
